# Supplementary material for: Association between oral microbiome and seven types of cancers in East Asian population: a two-sample Mendelian randomization analysis
Source: Front Mol Biosci. 2023 Nov 21;10:1327893. doi: 10.3389/fmolb.2023.1327893 (PMC10702768; doi:10.3389/fmolb.2023.1327893)
Supplement: Supplementary file 1 [file DataSheet1.ZIP › Supplementary Materials/MR plots of saliva/saliva═╝/colorectal cancer/pheno.2986_to_colorectal cancer_forest.pdf]

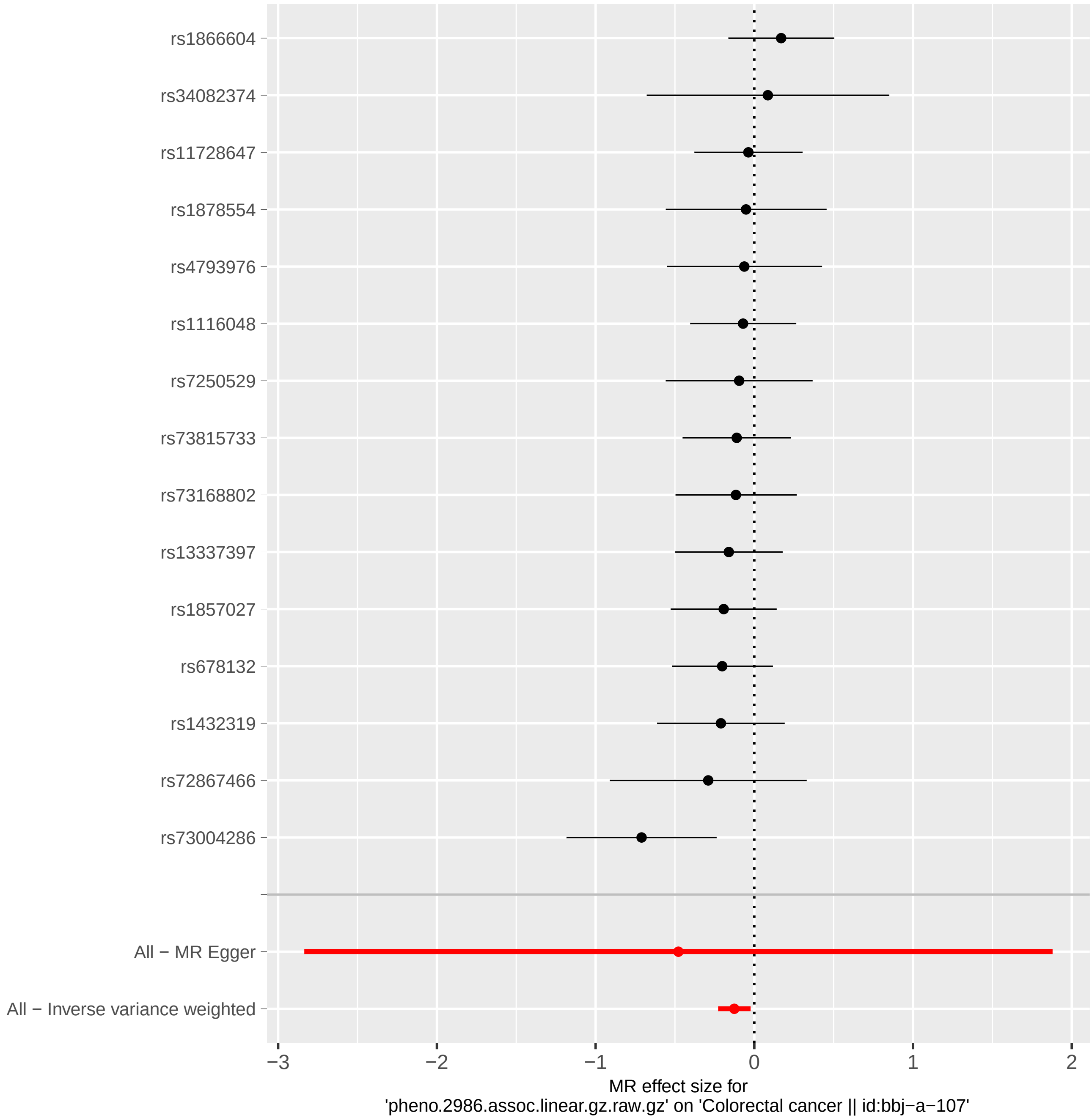
rs1866604
rs34082374
rs11728647
rs1878554
rs4793976
rs1116048
rs7250529
rs73815733
rs73168802
rs13337397
rs1857027
rs678132
rs1432319
rs72867466
rs73004286
All − MR Egger
All − Inverse variance weighted
−3
−2
−1
0
1
2
MR effect size for
'pheno.2986.assoc.linear.gz.raw.gz' on 'Colorectal cancer || id:bbj−a−107'
